# Comparative evaluation of Latanoprostene Bunod, Timolol Maleate, and latanoprost Ophthalmic Solutions to assess their safety and efficacy in lowering intraocular pressure for the management of Open-Angle Glaucoma

**DOI:** 10.6061/clinics/2020/e1874

**Published:** 2020-11-25

**Authors:** Yulong Wang, Yue Liao, Xin Nie

**Affiliations:** I Department of Ophthalmology, Chongqing General Hospital, Chongqing, China; II Department of Pharmacy, Chongqing General Hospital, Chongqing, China

**Keywords:** Intraocular Pressure, Latanoprost, Latanoprostene Bunod, Open-Angle Glaucoma, Timolol

## Abstract

**OBJECTIVES:**

Timolol maleate has been reported to be a safer intraocular pressure (IOP) lowering treatment than latanoprost. The United States Food and Drug Administration approved latanoprostene bunod, a nitric oxide-donating prodrug of latanoprost, for lowering IOP. This study compared the safety and efficacy of latanoprost, latanoprostene bunod, and timolol maleate in patients with open-angle glaucoma.

**METHODS:**

Patients who received latanoprost eye drops once daily in the evening were included in the latanoprost Ophthalmic Solutions (LP) cohort (n=104). Those who received latanoprostene bunod eye drops once daily in the evening were included in the Latanoprostene Bunod (LB) cohort (n=94). Those who received timolol eye drops twice daily were included in the Timolol Maleate (TM) cohort (n=115). All treatments were administered to the affected eye(s) for 3 months. Informed Consent has been taken from each participant before the trial.

**RESULTS:**

At the end of 3 months of treatment, latanoprost, latanoprostene bunod, and timolol were all successful in reducing IOP. The LB cohort had the highest reduction in IOP, compared to the LP and TM cohorts. All treatments had some common adverse ocular effects.

**CONCLUSION:**

Latanoprostene bunod was superior to latanoprost and timolol for the treatment of open-angle glaucoma.

## INTRODUCTION

Glaucoma is the third leading cause of irreversible blindness worldwide. Open-angle glaucoma may lead to permanent blindness (1,2). Elevated intraocular pressure (IOP) is responsible for glaucoma, and most treatments are designed to reduce IOP (3). Worldwide, approximately 80 million people have been predicted to have glaucoma by the end of 2020, with 11 million being bilaterally blind. While half of the population with glaucoma in high-income countries is unaware of their disease, this figure is over 90% in low-income countries, particularly in the rural settings (4).

The ultimate goal of glaucoma treatment is to slow down disease progression to a rate in which the patient will not experience a vision-related decrease in quality of life (5,6). Glaucoma treatment in developing countries should consider clinical, laser, and surgical approaches. Glaucoma medications do not improve vision, may have important side effects, and are relatively expensive. Thus, compliance can be a major issue, which is related to the level of education and socio-economic status of the patient (5,6).

Latanoprost, a Prostaglandin F2 alpha (PGF2α) analog, was approved by the United States Food and Drug Administration (USFDA) in 1996 (4). Latanoprost is a prostanoid selective FP receptor agonist that is believed to reduce IOP by increasing the outflow of the aqueous humor. Studies have suggested that the main mechanism of action is increased uveoscleral outflow. The higher the level of IOP, the greater is the likelihood of the optic nerve damage and visual field loss (4). The mechanism of action of timolol is through reduction in formation of the aqueous humor in the ciliary body in the eye. It was the first beta-blocker approved for topical use in the treatment of glaucoma in the US (1978) (7).

In November 2017, the USFDA approved latanoprostene bunod ophthalmic solution, a nitric oxide-donating prodrug of latanoprost, for lowering IOP(4). Latanoprostene bunod is hydrolyzed by corneal esterases to the prostanoid FP receptor agonist latanoprost acid (active metabolite) and butanediol mononitrate, which is further metabolized to 1.4-butanediol and nitric oxide (NO) (active metabolite) (8). Latanoprost acid increases matrix metalloproteinase (MMP)-1, MMP-3, and MMP-9 expression in the ciliary muscles, promoting the remodeling of the extracellular matrix and, subsequently, increases aqueous humor outflow through the uveoscleral pathway (8,9). The efficacy of timolol ophthalmic solution in the reduction of IOP has also been reported (10,11).

A preclinical study reported that latanoprostene bunods have an IOP-lowering effect in patients who do not respond to latanoprost (12). However, IOP-lowering treatments have safety issues. Timolol has been reported to be safer than latanoprost (13). Therefore, this study retrospectively compared the efficacy and safety of latanoprost, latanoprostene bunod, and timolol in patients with ocular hypertension or primary open-angle glaucoma.

## MATERIAL AND METHODS

### Ethics approval and consent to participate

The study protocol was approved by the Medical Council of China and local institutional review board (PHU/CL/15/19 dated October 5, 2019). Informed consent was obtained from each participant before the trial. Data regarding treatment and follow-up of patients were collected from the hospitals for analysis after obtaining approval.

### Study population

**Figure 1:**
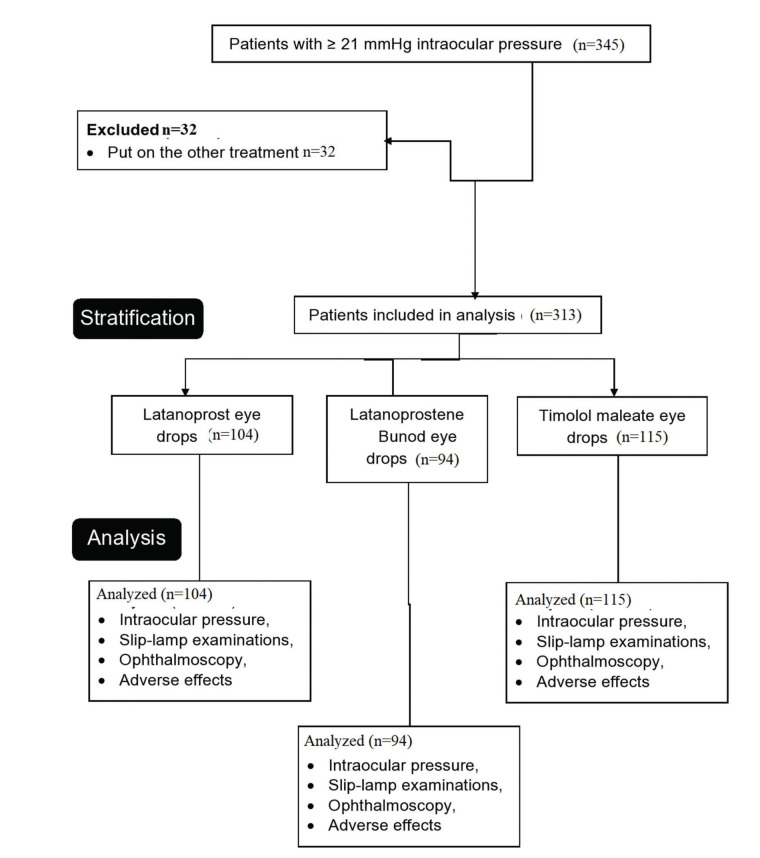
Flow chart of the study.

From February 15, 2018 to July 12, 2019, 345 patients were seen in the outpatient setting of the parent hospital and referring hospitals with an IOP of ≥21 mmHg. Among them, 104 patients were prescribed latanoprost, 94 patients with latanoprostene bunod, and 115 patients were prescribed timolol maleate for lowering IOP. The data on treatment and follow-up of these 313 patients were included in the analysis (Fig. 1).

### Cohorts

Patients who received 0.005% w/v latanoprost eye drops once daily in the evening in the affected eye(s) for 3 months were included in the LP cohort (n=104). Those who received 0.0024% w/v latanoprostene bunod eye drops once daily in the evening in the affected eye(s) for 3 months were included in the LB cohort (n=94). Those who received 0.5% w/v timolol maleate eye drops twice daily (morning and evening) in the affected eye(s) for 3 months were included in the TM cohort (n=115).

#### Exclusion criteria

1. History of hypersensitivity or contraindications to latanoprost, NO-donating medications, timolol maleate, other β-adrenergic receptor antagonists, or any ingredients in the study drugs
2. Significant corneal surface abnormalities in either eye or other significant ophthalmic disease
3. Patients requiring treatment with ocular or systemic corticosteroids or had an anticipated need to initiate or modify medication that was known to affect IOP
4. Patients who did not want to fill the informed consent and could not be followed-up

### IOP measurements

IOP was measured in the morning or evening, and the average of three readings was considered for analysis.

### Safety measurements

Safety measurements included slip-lamp examinations, ophthalmoscopy, and treatment-emergent unwanted issues during the 3 months of treatment.

#### Standardization regarding sleep and medication:

Before the 24-h laboratory recording, the patients were instructed to maintain 8 h of regular sleep every day, for a minimum of 7 days. Sleep patterns were verified using a wrist monitor for light exposure, arm movements, and a wake/sleep log. Patients were asked to abstain from alcohol for a minimum of 7 days and regular coffee for a minimum of 2 days before reporting to the laboratory. The 8-h sleep time for each patient in the laboratory was adjusted close to the recorded bedtime in the previous week, and this period was referred to as the nocturnal/sleep period.

#### Measurement of IOP and blood pressure:

Measurements of IOP and blood pressure/heart rate were assessed by experienced physicians. IOP was measured using a calibrated pneumatonometer. Topical proparacaine 0.5% was used as the local anesthetic. Each plot of IOP measurement by the pneumatonometer was evaluated according to commonly accepted standards. Blood pressure and heart rate were measured immediately before IOP measurements using an automated arm monitor.

#### Evaluation of adverse effects:

Ocular adverse effects were recorded by asking questions, visual inspections, and using the appropriate instruments as applicable. Systematic adverse effects were evaluated by measuring systolic and diastolic blood pressures and heart rate. Normal systolic and diastolic blood pressure values were 160 and 90 mmHg, respectively. The normal heart rate was considered as 80 beats per min (bpm).

### Statistical analyses

SPSS version 25 (IBM Corporation, Armonk, NY, USA) was used for statistical analysis. Fischer’s exact test was performed for ordinal data, and one-way analysis of variance (ANOVA) was performed for numerical data. The Tukey’s test was performed for post hoc analysis. The results were considered significant at the 95% confidence level.

## RESULTS

### Enrollment

The study patients’ ages ranged from 45 to 65 years, and the male: female ratio was 1:1. Other demographic parameters and clinical conditions are reported in Table 1. There were no significant differences among the cohorts at the time of initial diagnosis (*p*>0.05).

### Efficacy

**Figure 2:**
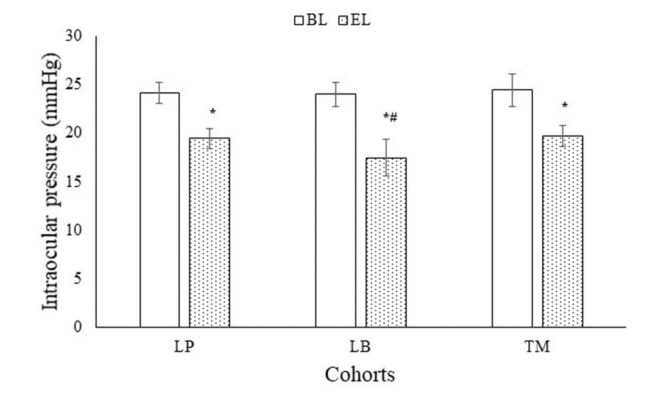
Intraocular pressure measurements. Data are represented as mean±SD. One-way ANOVA following the Tukey test was performed for statistical analysis. *significantly lower than BL. #Significantly lower than LP cohort and TM cohort. BL: Before treatment; EL: At the end of 3 months of treatment; SD: standard deviation; ANOVA: analysis of variance; LP: those who received latanoprost eye drops; LB: those who received latanoprostene bunod eye drops; TM: those who received timolol eye drops *p*<0.05 and I>3.336 were considered significant.

At the end of 3 months of treatment, all the enrolled drugs showed a significant decrease in IOP. After 3 months of treatment, latanoprost (24.13±1.12 mmHg *vs*. 19.45±1.01 mmHg, *p*<0.0001, q=42.749), latanoprostene bunod (23.98±1.22 mmHg *vs*. 17.45±1.89 mmHg, *p*<0.0001, q=43.945), and timolol (24.39±1.65 mmHg *vs*. 19.68±1.08 mmHg, *p*<0.0001, q=38.404) were all successful in the reduction of IOP. The LB cohort had the highest reduction in IOP compared to the LP (*p*<0.0001, q=14.654) and TM cohorts (*p*<0.0001, q=16.723, Fig. 2).

### Safety

All treatments had some common adverse ocular effects. The total ocular adverse effects reported in the TM cohort were less than those in the LP and LB cohorts *(p*=0.031, Table 2).

**Figure 3:**
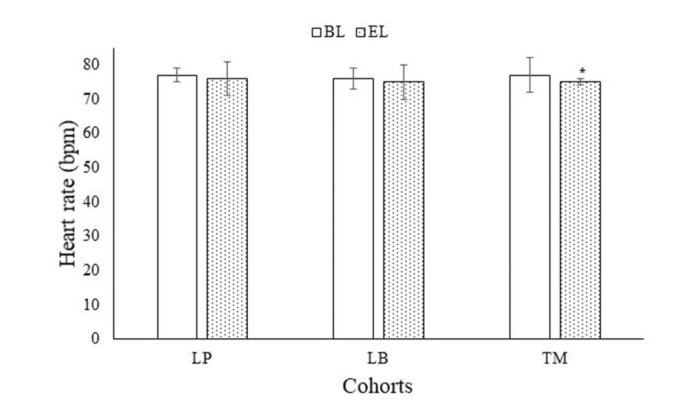
Heart rate evaluation. Data are represented as mean±SD. One-way ANOVA following the Tukey test was performed for statistical analysis. *significantly lower than BL. BL: Before treatment; EL: At the end of the 3 months of treatment; SD: standard deviation; ANOVA: Analysis of variance; LP: those who received latanoprost eye drops; LB: those who received latanoprostene bunod eye drops; TM: those who received timolol eye drops *p*<0.05 and q>3.336 were considered significant.

At the end of 3 months of treatment, timolol showed a decline in heart rate; however, latanoprostene bunod and latanoprost did not affect the heart rate (Table 3). After 3 months of treatment, latanoprost (77±2 bpm *vs*. 76±5 bpm, *p*=0.059) and latanoprostene bunod (76±3 bpm *vs*. 75±5, *p*=0.0002, q=2.789) did not decrease heart rate; however, timolol (77±5 bpm *vs*. 75±1 bpm, *p*<0.0001, q=6.776) reduced heart rate (Fig. 3).

The mean percentage changes from baseline were similar in all three treatment groups for corneal thickness (1.15±0.03, 1.16±0.11, and 1.14±0.08, *p*=0.193).

## DISCUSSION

Latanoprost, latanoprostene bunod, and timolol were all successful in reducing IOP after 3 months. Latanoprostene bunod produced a greater reduction in IOP than latanoprost and timolol. Therefore, this study showed that the efficacy of latanoprostene bunod was comparatively higher than latanoprost and timolol, with latanoprostene bunod reaching the target of ≤17.5 mmHg (14). The results of the study were consistent with previous randomized, double-blinded studies (1,6) and a preclinical study (12). Pharmacological therapies in open-angle glaucoma include PG analogs or *β*-blockers (15). Latanoprostene bunod is a PG F2a analog with NO-donating moiety and reduces IOP through the trabecular meshwork (16) and uveoscleral outflow pathway. Latanoprostene bunod is more effective in the treatment of open-angle glaucoma than latanoprost and timolol.

This study reported higher ocular adverse events for latanoprostene bunods than for timolol. The ocular adverse events were comparable to those of the randomized, double-blinded studies (1,6) and randomized crossover clinical trial (17). Further research is required to decrease ocular adverse effects related to latanoprostene bunod treatment in open-angle glaucoma.

Unlike latanoprost and latanoprostene bunods, timolol eye drops decreased heart rate and systolic and diastolic blood pressure at the end of 3 months of treatment. The results of these non-ocular adverse events were not comparable with the randomized, double-blinded studies (1,6); however, they were comparable with a prospective study (18) and randomized crossover clinical trial (17). Timolol is a *β*-blocker and administration for 3 months led to a systemic effect and decrease in heart rate and blood pressure (18).

In 2016, Weinreb et al. reported that latanoprostene bunod 0.024% demonstrated a significantly greater lowering of IOP than timolol 0.5% administered twice daily over 3 months of treatment (19).

Medeiros et al. concluded in 2016 that the administration of latanoprostene bunod 0.024% daily in the evening was noninferior to timolol 0.5% twice daily over 3 months of treatment, with significantly greater IOP-lowering in patients with open-angle glaucoma or ocular hypertension at all but the earliest time point evaluated, and demonstrated a good safety profile (20).

This study has some limitations. As a retrospective study, there were chances of bias in the selection of patients. A large double-blind placebo-controlled randomized trial is required to explore this hypothesis unambiguously. Patients’ demographic and clinical conditions can also affect the adverse events during the treatment period; however, the study did not evaluate the effects of such parameters on clinical outcomes.

## CONCLUSIONS

Because of its novel mechanism of action, latanoprostene bunod was more successful in reducing IOP in patients with open-angle glaucoma than latanoprost and timolol, with manageable adverse events. Latanoprostene bunod is superior to latanoprost and timolol in the treatment of open-angle glaucoma.

Future Implication: Large ample size prospective studies are needed to validate that latanoprostene bunod 0.024% medication has the potential to be used as a first-line therapy for patients to reduce IOP, decrease the risk of glaucoma progression, and preserve vision while maintaining their quality of life.

## AUTHOR CONTRIBUTIONS

Wang Y contributed in Conceptualization (Equal), Data curation (Equal), Investigation (Lead) Methodology (Equal), Project administration (Lead), Supervision (Lead), Writing-review & editing (Lead). Liao Y contributed in Conceptualization (Equal), Data curation (Equal), Formal analysis (Lead), Investigation (Equal), Methodology (Lead), Resources (Lead), Validation (Equal). Xin Nie X contributed in Conceptualization (Lead), Investigation (Equal), Software (Equal), Writing-original draft (Lead).

## Figures and Tables

**Table 1:** Other demographic parameters and clinical conditions of the stratified patients.

Cohort		LP	LB	TM	Comparison between cohorts
Eye drop		0.005% latanoprost	0.0024% latanoprostene bunod	0.5% timolol	
Patients		104	94	115	*p*-value
Age (years)	Minimum	45	45	46	0.684
	Maximum	65	64	65	
	Mean±SD	58.42±6.12	57.65±6.01	57.99±6.44	
Sex	Male	55 (53)	49 (52)	53 (46)	0.544
	Female	49 (47)	45 (48)	62 (54)	
Family history		9 (9)	7 (7)	8 (7)	0.891
Ethnicity	Han Chinese	93 (89)	85 (90)	102 (89)	0.994
	Mongolian	10 (10)	8 (9)	12 (10)	
	Tibetan	1 (1)	1 (1)	1 (1)	
Intraocular pressure (mmHg)		24.13±1.12	23.98±1.22	24.39±1.65	0.089
Heart rate (bpm)		77±2	76±3	77±5	0.085

Ordinal data are presented as number (percentage), and numerical data are presented as mean±SD.

Fischer’s exact test was performed for ordinal data, and one-way ANOVA was performed for numerical data. *p*<0.05 was considered significant.

SD: Standard deviation; LP: those who received latanoprost eye drops; LB: those who received latanoprostene bunod eye drops; TM: those who received timolol eye drops.

**Table 2:** Ocular adverse events.

Cohort	LP	LB	TM	Comparison between cohorts
Eye drop	0.005% latanoprost	0.0024% latanoprostene bunod	0.5% timolol	
Patients	104	94	115	*p*-value
Eye irritation	4 (4)	4 (4)	2 (2)	0.529
Dry eye	2 (2)	2 (2)	1 (1)	0.731
Eye pain	3 (3)	3 (3)	1 (1)	0.445
Conjunctival hyperemia	4 (4)	3 (3)	1 (1)	0.340
Foreign body sensation in eye (s)	3 (3)	2 (2)	1 (1)	0.546
Total	16 (16)	14 (14)	6 (6)*	0.031

Data are presented as number (percentage).

The Fischer’s exact test was performed for statistical analysis.

*p*<0.05 was considered significant.

* Significantly lower than the LP and LB cohorts.

LP: those who received latanoprost eye drops; LB: those who received latanoprostene bunod eye drops; TM: those who received timolol eye drops.

**Table 3:** Effects on blood pressure.

Cohort	LP			LB			TM			
Eye drop	0.005% latanoprost			0.0024% latanoprostene bunod			0.5% timolol			
Level	BL	EL		BL	EL		BL	EL		
Patients	104	104	# *p*	94	94	# *p*	115	115	# *p*	#q
Diastolic blood pressure (mmHg)	83±4	82±4	0.073	84±3	83±4	0.054	85±5	82±3*	<0.0001	7.875
Systolic blood pressure (mmHg)	123±4	122±5	0.113	125±5	124±4	0.132	127±4	123±2*	<0.0001	12.452

Data are presented as mean±SD.

One-way ANOVA following the Tukey test was performed for statistical analysis.

# Between BL and EL.

* significantly lower than BL.

BL: Before treatment; EL: At the end of the 3 months of treatment; SD: Standard deviation; ANOVA: Analysis of variance; LP: those who received latanoprost eye drops; LB: those who received latanoprostene bunod eye drops; TM: those who received timolol eye drops.

*p*<0.05 and q>3.336 were considered significant.
